# A novel adjuvant drug-device combination tissue scaffold for radical prostatectomy

**DOI:** 10.1080/10717544.2019.1686085

**Published:** 2019-11-18

**Authors:** Ketevan Paliashvili, Francesco Di Maggio, Hei Ming Kenneth Ho, Sanjayan Sathasivam, Hashim Ahmed, Richard M. Day

**Affiliations:** a Centre for Precision Healthcare, UCL Division of Medicine, University College London, London, UK;; b UCL Chemistry Department, University College London, London, UK;; c Department of Surgery & Cancer, Division of Surgery, Imperial College London, London, UK;; d The Discoveries Centre for Regenerative and Precision Medicine, University College London, London, UK

**Keywords:** Prostate cancer, adjuvant chemotherapy, docetaxel, radical prostatectomy, thermally induced phase separation, microparticles

## Abstract

Prostate cancer is a leading cause of death in men and despite improved surgical procedures that aid tumor resection, the risk of recurrence after surgery as a result of positive resection margins remains significant. Adjuvant chemotherapy is often required but this is associated with toxicity. Improved ways of delivering highly toxic chemotherapeutic drugs in a more controlled and targeted manner after the prostate has been removed during surgery could reduce the risk of recurrence and avoid systemic toxicity. The aim of this study was to develop a novel drug-device combination tissue scaffold that can be used to deliver the chemotherapeutic agent, docetaxel, into the tissue cavity that is created following radical prostatectomy. The device component investigated consisted of highly porous, poly(dl-lactide-co-glycolide) microparticles made using thermally induced phase separation. A facile method was established for loading docetaxel with high efficiency within one hour. Sustained drug release was observed from the microparticles when placed into a dynamic system simulating tissue perfusion. The drug released from the microparticles into perfusates collected at regular time intervals inhibited colony formation and exhibited sustained cytotoxicity against 3D spheroids of PC3 prostate cancer cells over 10 days. In conclusion, this study demonstrates the concept of combining docetaxel with the biodegradable microparticles at the point of care is technically feasible for achieving an effective drug-device combination tissue scaffold. This approach could provide an effective new approach for delivering adjuvant chemotherapy following radical prostatectomy.

## Introduction

Prostate cancer is one of the most prevalent cancers among men and a leading cause of mortality (Taitt, 2018; Miller et al., 2019). Radical prostatectomy is a standard treatment option for cancer that is confined to the prostate gland and can be performed via either minimally invasive surgery (laparoscopic or robotic techniques) or an open approach (Nelson et al., 2017; Cao et al., 2019).

A positive surgical margin (defined as the histological presence of cancer cells at the inked margin of the excised specimen) following radical prostatectomy is associated with a higher risk of biochemical recurrence due to residual tumor left in the patient (Karakiewicz et al., 2005; Swindle et al., 2005; Zhang et al., 2018). Although a more aggressive approach to surgical resection is more likely to obtain clear margins during prostatectomy, it is associated with a higher risk of urinary incontinence and erectile dysfunction due to collateral tissue damage (Haglind et al., 2015). Obstacles that may affect the goal of achieving clear surgical margins include anatomical factors and surgical technical skills (Haglind et al., 2015; Walz et al., 2016). It is therefore unsurprising that despite being a curative treatment for localized disease, around 50% of patients with high-risk prostate cancer will develop biochemical recurrence following surgery within 5 years and the associated with poor prognosis (Briganti et al., 2015). This outcome is often related to local recurrence either from positive surgical margins or shedding of cancer cells into the peri-prostatic space during extirpative surgery. For instance, even in intermediate risk cases, the incidence of positive surgical margins arising in laparoscopic radical prostatectomy and robotic-assisted radical prostatectomy is reported at ∼22% (Huang et al., 2017). Many of these patients will require adjuvant or salvage (radiation) therapy or systemic hormonal therapy, which carry higher risks of functional impact on erectile function or urinary continence. Men who then fail these therapies will need third line hormonal or chemotherapy drugs that confer higher risk of toxicity. Since it is not possible to completely excise all of the tumor or residual cancerous cells with 100% certainty, new multimodal treatments that safely enable the targeted delivery of an anti-cancer drug during surgery straight after the prostate is removed might be beneficial to improve outcomes and reduce risk of recurrence and requirement for further therapy.

Docetaxel, a taxane-based chemotherapy, binds to intracellular β-tubulin where it inhibits microtubule depolymerization resulting in mitotic arrest and activation of signaling pathways associated with apoptosis (Herbst & Khuri, 2003). In addition to its use as a first-line treatment option for metastatic castration-resistant prostate cancer (Attard et al., 2016; James et al., 2016a,b; Vale et al., 2016), early use of docetaxel (DTX) with radical prostatectomy in clinically localized, high-risk prostate cancer may improve recurrence-free survival when used as an adjuvant or neoadjuvant chemotherapeutic (Dreicer et al., 2004; Thalgott et al., 2014; Lin et al., 2016). However, its systemic use without hormonal therapy has shown no significant improvement in biochemical disease-free survival after radical prostatectomy (Ahlgren et al., 2018).

Like many anti-cancer drugs, clinical efficacy and improved survival with systemic delivery of taxanes is often associated with numerous drug-related toxicities due to nonspecific distribution required to achieve the therapeutic effect, with only a small fraction of the drug reaching the tumor (Brewer et al., 2016; Sibaud et al., 2016; Chiu et al., 2017). The anatomical accessibility of the prostate makes direct drug targeting a feasible possibility, especially with the advent of laparoscopic and robotic techniques. However, modeling of docetaxel delivery directly into the prostate has shown it is unlikely to be efficacious since directly delivered infusions readily pass into the urethra due to regions of tissue with high fluid conductivity (Raghavan et al., 2016). To achieve localized targeted delivery and to mitigate unwanted side-effects associated systemic delivery of taxanes, various polymer-based drug delivery systems have been investigated with the intention of achieving high therapeutic concentrations of chemotherapy at the site of malignant disease. These include nano-materials loaded with taxanes comprising of polymer nanoparticles (Bowerman et al., 2017), liposomes (Pereira et al., 2016), dendrimers (Rompicharla et al., 2019), and nanotubes (Niezabitowska et al., 2018). It is worth noting that many nanoscale materials devised to date for anti-cancer drug delivery rely on intravenous administration and therefore require mechanisms capable of targeting the drug to the tumor tissue at therapeutic concentrations. Moreover, their entry into the systemic circulation is likely to result in removal and sequestration by the reticuloendothelial system and off-target tissue accumulation, both of which are significant issues that require attention when developing materials in the nanoscale range. These concerns can be allayed in part by using controlled-release drug-delivery depot systems, such as films, wafers, gels, and particles, to provide localized controlled and sustained drug release when implanted into or adjacent to the tumor, thus increasing the likelihood of clinical efficacy and reducing off-target collateral tissue damage (Wolinsky et al., 2012).

Careful consideration of the target product profile to meet end user requirements opens up new opportunities and challenges for innovative drug-delivery combination products. For example, during radical prostatectomy, the site of tumor excision and thus any residual tumor cells is accessible. Therefore, a novel approach for adjuvant therapy could involve administering a drug delivery depot system at the time of tumor excision. Key attributes of such a depot system intended to destroy residual cancer cells that remain after excision of the prostate tumor include: (i) being capable of minimally invasive delivery through suitable gauge needle or delivery port; (ii) conformable to the shape of the tissue cavity that is created following excision of the tumor, so as to maximize the surface area of the medicinal product in close proximity to residual tumor cells; (iii) sustained delivery of a therapeutic dose; (iv) retention of the product at the target site; and (v) ideally translatable for clinical use within a short time-frame.

In this study, we aimed to develop a novel controlled-release drug-delivery depot system that meet these target attributes by combining the anti-cancer drug docetaxel with an existing, clinically approved class III medical device consisting of biodegradable highly porous microparticles made via thermally induced phase separation (TIPS). The TIPS microparticles are intended to provide an injectable scaffold that can be delivered immediately after excision of the prostate tumor that result in the microparticles conforming to the shape of the tissue cavity. The size of the microparticles will allow minimally invasive delivery but also create interstices between the packed microparticles for the infiltration of host tissue during healing following surgery. Loading of the anti-cancer drug onto the surface of the microparticles is intended to provide immediate localized release of an efficacious concentration of the drug that can effectively destroy residual tumor cells left following prostatectomy. To demonstrate proof of concept for this approach, the objectives of the study included first establishing a facile and robust loading regime that achieved consistent amounts of docetaxel loaded onto the TIPS microparticles that is compatible with minimally invasive delivery at the time of surgery, and second, *in vitro* efficacy of the docetaxel-TIPS microparticles combination product.

## Materials and methods

### Fabrication of TIPS microparticles

TIPS microparticles composed of poly(d,l-lactide-co-glycolide) (PLGA) were prepared as previously described (Ahmadi et al., 2011). PLGA PURASORB 7507 (75:25) polymer (Corbion, Amsterdam, Netherlands) was dissolved in dimethyl carbonate (Sigma-Aldrich, Dorset, UK) overnight using magnetic stirring to produce a 10% (w/v) polymer solution. The polymer solution then was fed into a Nisco Encapsulator Unit (Nisco Engineering, Zurich, Switzerland; frequency: 2.75 kHz, amplitude: 70%) by a syringe pump (Harvard Apparatus, Kent, UK), at a constant flow rate of 2 mL/min. The polymer droplets were formed using a 100 µm sapphire nozzle and collected in liquid nitrogen. Residual solvent was removed from the frozen polymer droplets by lyophilization for 48 h. The dried PLGA TIPS microparticles were sieved to a size range of 250–350 µm and stored at room temperature in rubber stoppered glass vials under vacuum.

### Loading of docetaxel with TIPS microparticles

A facile method was developed to load DTX onto TIPS microparticles via anti-solvent precipitation. Five milligrams of PLGA TIPS microparticles were transferred into 20 mL clear Type 1B borosilicate glass vials and sealed with a butyl injection stopper. 4.5 mL of ultrapure water was added to the vial and vortexed for 10 seconds. 0.5 mL of 0.1% (w/v) docetaxel in ethanol was added using a 1 mL syringe with a 25 G needle through the rubber stopper, when the vial was inverted. The vial was then vortexed for 10 seconds and placed on a roller mixer (IKA^®^ Roller 6 Digital; 60 rpm) at room temperature for predetermined periods (5, 15, 30, 60, and 120 minutes).

Docetaxel loading efficiency (DLE) onto the TIPS microparticles at each time-point was calculated according to the following equation (Miller et al., 2019). The amount of free docetaxel left in the solution was measured by UV spectroscopy at the wavelength of 229 nm using a Nanodrop 2000c spectrophotometer (Thermo Scientific, Waltham, MA).

(1) $$\mathrm{DLE}\;\left(\%\right)=\frac{\text{total DTX added}-\text{free DTX}}{\text{total DTX added}}\times 100\%$$

Scanning electron microscopy was used to investigate the morphological changes on the surface of TIPS microparticles upon the loading of docetaxel. Unbound docetaxel was removed from the microparticles by washing thrice with 5 mL ultrapure water, followed by desiccation under vacuum. Samples of the dried particles were coated with gold for 60 seconds using a Q150R ES gold coater (Quorum Technologies, Oxford, UK). The samples were imaged using a Hitachi S3400N scanning electron microscope.

X-ray photoelectron spectroscopy (XPS) was performed using a Thermo Scientific K-alpha photoelectron spectrometer (Waltham, MA) using monochromatic Alkα radiation. Higher resolution scans were recorded for the principal peaks of N(1s), C(1s) at a pass energy of 50 eV.

### Docetaxel release from the DTX-TIPS microparticles combination product

Release profile of DTX from the TIPS microparticles was investigated using a dynamic perfusion system to simulate tissue perfusion of the drug-device combination in the physiological milieu when used in clinic following radical prostatectomy. Thus, the perfusion system was placed inside an incubator at 37 °C and physiological simulated medium (phosphate-buffered saline (PBS); pH 7.4) was used as perfusate. DTX-loaded microparticles were mixed with 100 µL of 70% (v/v) GranuGel^®^ (Convatec, Reading, UK) diluted in ultrapure water and the mixture was placed between two 25 mm circular filter papers (Whatman^®^ qualitative cellulose filter paper, Grade 1), where their positions were held by a Swin-Lok™ plastic membrane filter holder. A hypodermic needle (18 G × 40 mm) connecting the outlet of the filter holder, was inserted through the lid of a 50 mL polypropylene container to collect the perfusate. PBS was pumped through the filter holder by a peristaltic pump (Harvard Apparatus, Cambridge, MA) at a flow rate of 0.01 mL/min. The conditioned perfusate was sampled at the specified intervals and used for further experiments.

The amount of DTX released in the perfusate was determined using a predetermined standard curve. At each measurement, the concentration of DTX in the release medium collected in the polypropylene container was determined by UV spectroscopy at 229 nm as described above, to calculate the cumulative DTX release according to the following equation (Taitt, 2018).

(2) $$\text{Cumulative release }\left(\%\right)=\;\frac{\text{concentration of DTX in perfusate }\times \text{ total volume of perfusate}}{\text{total DTX loaded on the TIPS}}\times 100\%$$

### *In vitro* efficacy of docetaxel released from the DTX-TIPS microparticles combination product

Human prostate cancer cells (PC3, American Type Culture Collection, Manassas, VA) were used to test the activity of the docetaxel released from the DTX-TIPS microparticles. PC3 cells were maintained in Ham’s F12-K medium (Kaighn’s modification) (Invitrogen, Carlsbad, CA, 21127-022) supplemented with 10% (v/v) fetal bovine serum (FBS) and 1% antibiotics (referred to henceforth as complete medium). Cells were cultured at 37 °C under 5% CO_2_ atmosphere in a humidified incubator.

PC3 cells cultured in six-well plates were incubated for 48 hours in 2 mL of perfusate (conditioned complete culture medium) collected from DTX-TIPS microparticles loaded into the perfusion system, as outlined above. Colony formation assays were performed as previously described (Franken et al., 2006). The PC3 cells exposed to the perfusate were detached from the six-well plates using trypsin-EDTA solution (0.5 g/L porcine trypsin and 0.2 g/L EDTA.4Na in Hank's Balanced Salt Solution with phenol red; Sigma-Aldrich, Dorset, UK), washed in fresh complete medium to produce a single cell suspension, resuspended in fresh complete medium and re-plated into 9 cm diameter petri dishes at a density of 400 cells per dish. After plating, the dishes were incubated at 37 °C under 5% CO_2_ atmosphere in a humidified incubator for 2 weeks to allow formation of colonies. The colonies were fixed with methanol for 20 min and stained with 0.5% crystal violet (Sigma-Aldrich, Dorset, UK) in distilled water for 2 h. The colonies were counted in ImageJ using the ‘Colony Counter’ plug in (processing parameters: size 100–30,000; circularity 0.5–1) (https://imagej.nih.gov/ij/plugins/colony-counter.html).

Plating efficiency (PE) was estimated by dividing the number of colonies counted by the number of cells initially seeded. This number was used for normalization in calculating the surviving fraction (SF) (Franken et al., 2006).

(3) $$\mathrm{SF}=\frac{\text{number of colonies formed after treatment}}{\text{number of cells seeded }\times \;\mathrm{PE}}$$

### Morphological detection of apoptosis in 2D culture

Morphological changes to PC3 cells indicative of apoptosis, including shrinkage and fragmentation into membrane-bound apoptotic bodies, were assessed following exposure to perfusate conditioned complete medium. Cells were incubated for 24 hours in the perfusate conditioned complete medium collected over 12 days at 24-hour intervals. Images of cell morphology were acquired using phase contrast microscopy using a Zeiss Primovert microscope (Oberkochen, Germany) and at least 100 cells in each group were analyzed to calculate the number of cells in each image displaying nuclear fragmentation.

### Efficacy studies with tumor spheroids

3D spheroids of PC3 cells were generated by using methylcellulose as a scaffold, as previously described (Burris et al., 1993). PC3 were seeded at a concentration of 2 × 10^4^ cells/200 µL complete culture medium containing 20 wt% methylcellulose in 96-well ultralow attachment u-bottom plates. The cells were incubated for two days at 37 °C under 5% CO_2_ atmosphere in a humidified incubator until the spheroids had formed. The medium was replaced with 200 µL perfusate conditioned complete medium that was replaced daily with medium collected from the perfusate system at the corresponding time-point over 12 days.

Images of the spheroids were acquired for each day of the culture. The dimensions of the imaged spheroids were measured using Image J (Bethesda, MD). Feret’s diameter was used to estimate the mean diameter of spheroids and plotted against time (GraphPad Prism Version 8.0; GraphPad Software, San Diego, CA).

### Statistical methods

Data were tested for the Gaussian distribution and analyzed for statistical significance using GraphPad Prism software (GraphPad Software, San Diego, CA). For data sets with a Gaussian distribution, statistical evaluation was performed by two-way ANOVA with Dunnett’s test for multiple comparisons unless stated otherwise in the figure legend. For data sets with a non-Gaussian distribution, statistical evaluation was performed by the Friedman test.

## Results

### Immobilization of DTX onto PLGA TIPS microparticles

**Figure 1.**
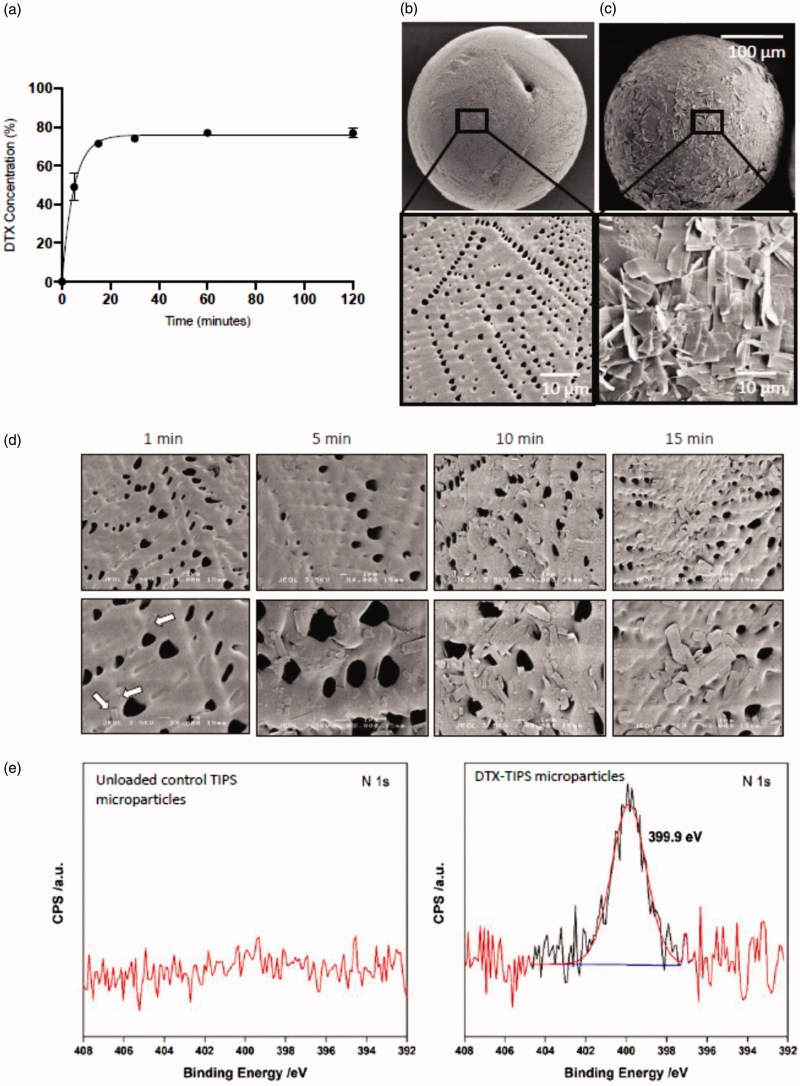
Immobilization of DTX onto PLGA TIPS microparticles. (a) The amount of DTX loaded onto the microparticles was indirectly quantified by calculating the amount of DTX remaining in solution (measured by UV absorption) at different time points during the loading phase. Representative scanning electron microscopy (SEM) images of (b) unloaded control TIPS microparticles and (c) DTX-TIPS microparticles loaded with 500 µg of DTX. (d) SEM images demonstrating the time-dependent adsorption of DTX to the surface of TIPS microparticles. (e) XPS high-energy resolution spectra of nitrogen (N1s) obtained on the surface of the control TIPS microparticles or DTX-TIPS microparticles.

The dried PLGA TIPS microparticles initially floated at the top of the DTX solution but were constantly mixed with solution by rotation of the glass vial during the loading phase. The amount of DTX loaded onto the microparticles was indirectly quantified by calculating the amount of DTX remaining in solution at different time points during the loading phase. Approximately, 80% of the DTX was loaded onto the microparticles from the solution within 60 minutes of initiating mixing (Figure 1(a)). No further increase in loading was detected beyond 60 minutes during the loading phase. Scanning electron microscopy of the microparticles revealed the presence of crystalline material on the surface of the microparticles incubated in the DTX solution compared with unloaded control TIPS microparticles (Figure 1(b,c)). The crystalline material was visible on the surface of the microparticles within one minute of incubation and progressively increased with duration of incubation time (Figure 1(d)). The molecular composition of DTX (C_43_H_53_NO_14_) allowed for XPS analysis for nitrogen to be used to confirm the elemental composition of the crystalline material on the surface of the microparticles (Figure 1(e)). A strong nitrogen signal was detected on the DTX-loaded microparticles that was absent in the unloaded control microparticles.

### Cytotoxic activity of sustained DTX release against prostate cancer cells

**Figure 2.**
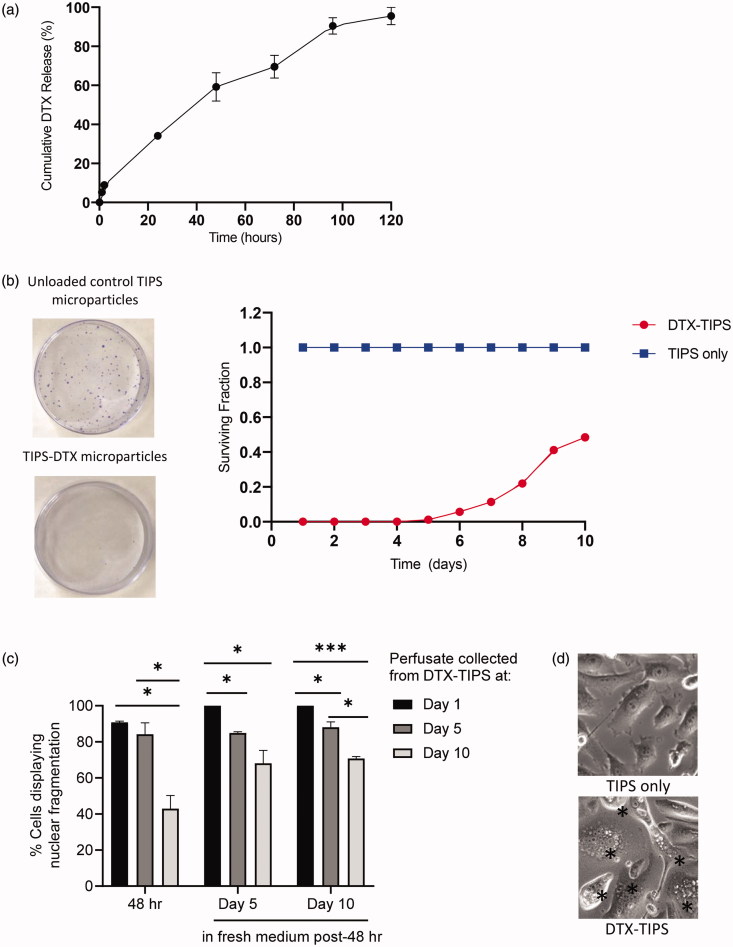
(a) Cumulative release of DTX from DTX-TIPS microparticles measured using UV absorption at 229 nm. (b) Colony formation assay performed by incubating PC3 cells for 48 hours with perfusate collected at daily intervals from DTX-TIPS microparticles over a period of 12 days. The cells exposed to perfusate collected from the different time points were re-plated into petri dishes and incubated for 2 weeks before the stained colonies were counted. The smaller surviving fraction of colonies treated with perfusate collected from DTX-TIPS at all time-points indicates the cytotoxic activity of the released drug. (c) PC3 cells treated with perfusate collected from DTX-TIPS exhibited morphological features associated with cytotoxicity. Quantification of cells displaying morphological changes associated with apoptosis following incubation of PC3 cells for 48 hours with perfusate collected DTX-TIPS microparticles at days 1, 5, and 10. Cell morphology was assessed at 48 hours post-incubation and at 5 and 10 days post-incubation in fresh complete medium replenished every 2 days (*p<.05, ***p<.001). (d) Photomicrographs of cells treated with perfusate collected from TIPS only microparticles or DTX-TIPS microparticles. Cells in the DTX-TIPS group display morphological features of apoptosis indicated by *.

The release profile of DTX from TIPS microparticles was investigated using a dynamic perfusion system designed to simulate tissue perfusion *in vivo* during the intended clinical use of the drug-device combination. The perfusate containing DTX from the TIPS microparticles was collected at regular intervals and the quantity of DTX measured using UV absorbance. A plot of the cumulative release of DTX shows approximately 95% of the total amount of DTX loaded onto the TIPS microparticles was released over five days, with approximately one-third being released during the first 24 hours (Figure 2(a)). The quantity of DTX released at time-points beyond five days was below the detection threshold for the system used for UV absorbance. Therefore, two cell-based assays using PC3 prostate cancer cells were used to assess whether sustained cytotoxic activity existed for DTX released from TIPS microparticles in the perfusate. The colony formation assay revealed DTX released into the perfusate from the TIPS microparticles collected from all time points up to day 10 continued to have an inhibitory effect on the formation PC3 cell colonies (Figure 2(b)). DTX present in the perfusate collected from days 1 to 4 completely suppressed the formation of all colonies. The formation of colonies after incubation in perfusates collected between days 5 and 8 was less than 25% of the number of colonies established in the control group containing no DTX. The number of colonies formed at day 10 was approximately 50% of the number of colonies established in the control group containing no DTX. Further confirmation of the sustained cytotoxic effect was revealed by morphological analysis of cells initially incubated in the DTX-TIPS perfusate for 48 hours followed by incubation in fresh complete medium for 1, 5, or 10 days (Figure 2(c)). Phase contrast microscopy revealed characteristic morphological features of apoptosis including nuclear fragmentation, cell rounding due to shrinkage and cytoplasm condensation (indicative of early apoptosis) and apoptotic bodies (indicative of later-stage apoptosis).

### Cytotoxic activity of sustained DTX release against prostate cancer spheroids

**Figure 3.**
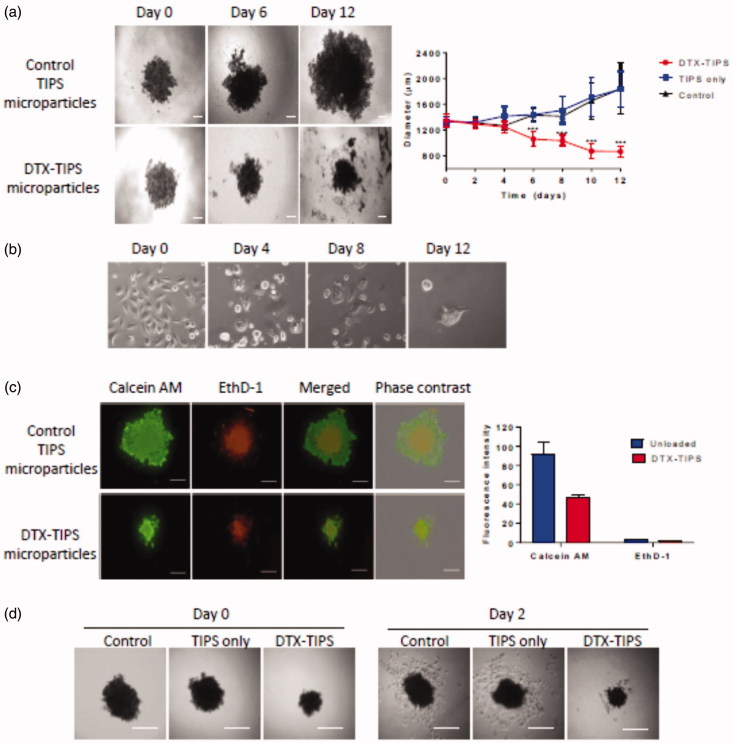
(a) 3D spheroid cultures of PC3 cells were incubated in perfusate collected from DTX-TIPS or unloaded control TIPS microparticles for 12 days. The culture medium was replaced with perfusate collected at the corresponding time points. The diameter and volume of the spheroids was calculated from images acquired throughout the incubation period. Spheroids incubated with perfusate from DTX-TIPS shrank in size over time. (b) PC3 cells cultured in 2D in tissue culture wells were treated in the same manner and exhibited morphological features of cytotoxicity. (c) The viability of cells in the spheroids incubated with perfusate at day 12 was analyzed using Live/Dead^®^ staining (calcein AM – live cells (green) and ethidium homodimer-1 (EthD-1) dead cells (red)) Scale bars 200 µm. (d) PC3 cells in spheroids incubated with perfusate from DTX-TIPS microparticles and transferred to tissue culture plates at day 12 failed to migrate from the spheroid and appeared non-viable, whereas cells from spheroids incubated with control microparticles were viable and migrated from the spheroid. Scale bar 200 µm. Data represent mean ± standard deviation from *n* = 20. ***p<.001 between control and DTX treated samples.

To assess the cytotoxicity of DTX released from the DTX-TIPS combination in a more physiological 3D culture system, 3D spheroids composed of PC3 cells were incubated with the perfusates collected daily over 12 days. The medium containing the spheroids was replaced on a daily basis with perfusate collected from the perfusion system on the corresponding day. The diameter and volume of the spheroids calculated from images acquired throughout the experiment showed a significant reduction in size compared with both the starting size of the spheroid and spheroids in control groups measured at the same time-points (Figure 3(a)) (PC3 cells cultured in 2D in tissue culture wells that were exposed to the perfusates collected daily over 12 days points exhibited morphological features of cytotoxicity similar to that observed in the colony formation assays (Figure 3(b))). Live/Dead^®^ staining with calcein AM (an enzymatic fluorescent dye that passively enters and stains all metabolically active cells) and ethidium homodimer-1 (EthD-1; a fluorescent dye that only stains dead cells by binding to nucleic acid after passing through their compromised membranes) was performed on the spheroids incubated with perfusate collected over a day 12 period (Figure 3(c)). Dead cells were visible towards the center of the spheroids in both the DTX treated and control spheroids indicating the presence of a necrotic core due to the diffusional limits of oxygen and nutrients. Exposure of the spheroid to DTX in the day 12 perfusate resulted in a reduction in the size of the spheroid diameter. PC3 cells in the remaining spheroid were stained positive for calcein AM and EthD-1 but the proportion of EthD-1 positive cells at the periphery of the spheroids exposed to DTX was increased compared with the control group. The cytotoxic effect of DTX released from the DTX-TIPS particles against the 3D spheroid cultures was confirmed by transferring the spheroids at day 12 to tissue culture plates. No viable cells migrating from the spheroids were visible in the DTX treated spheroids, whereas cells could be seen migrating from the spheroids in the control group.

## Discussion

A positive surgical margin following radical prostatectomy exists in a significant proportion of patients and is associated with a high risk of prostate cancer recurrence. Adjuvant chemotherapy using DTX after surgical resection has been advocated for many different types of solid tumor. Conventional intravenous routes of delivery increase the likelihood of DTX clearance, which is related to α1-acid glycoprotein level, hepatic function, age, and body surface area. When administered intravenously, the drug binds to α1-acid glycoprotein, lipoproteins, and albumin in the plasma (Kenmotsu & Tanigawara, 2015). The drug is metabolized by the hepatic cytochrome P450 (CYP) 3A isoforms CYP3A4 and CYP3A5, and the predominant route of elimination is via biliary and intestinal excretion (Shou et al., 1998; Baker et al., 2006). Such modes of clearance may account for the reported lack of benefit when DTX is given via conventional routes after radical prostatectomy (Ahlgren et al., 2018).

A localized delivery and sustained release formulation of the chemotherapeutic agent at the site of resected prostate gland would allow for increased local concentrations of the agent at the tumor site, potentially reducing the risk of disease recurrence arising from incomplete resection of the prostate cancer by destroying residual tumor cells. Moreover, a localized delivery system would reduce the risk of chemotherapeutic agent losses within the circulation and off-target toxicity resulting in neutropenia, leukopenia, neurological toxic effects, diarrhea, alopecia, asthenia, and nausea associated with docetaxel monotherapy delivered using conventional systemic delivery routes (Burris et al., 1993; Extra et al., 1993).

In the current study, we have investigated implantable, biodegradable microparticles for use as medical device in the sustained delivery of DTX. The medical device component (TIPS microparticles) of the drug-delivery system is designed to serve as a conformable scaffold that will readily fill the tissue cavity left by resected prostate. The microparticles are of a size range (250–350 µm) that can be easily delivered via a suitable gauge needle or delivery port via a minimally invasive approach while providing sufficiently large interstices between packed microparticles to increase the surface area for drug release and allow tissue infiltration between the close packed microparticles. These physical attributes differentiate the TIPS microparticles used in our drug-delivery system to those used in the PACLIMER^®^ microspheres delivery system, which incorporates paclitaxel into biodegradable microspheres with a median diameter of 53 μm (size range from 20 to 200 μm in diameter) composed of polyphosphoester polymer poly(d,l-lactide co-ethyl phosphate) (Harper et al., 1999). The smaller microspheres used with PACLIMER^®^ are not intended to pack tissue cavities such as that left after prostatectomy and would not provide interstices to allow tissue infiltration. Furthermore, the median size of PACLIMER^®^ microspheres is below the accepted migratory threshold of 80 μm that has been associated with the migration of microparticles from the site of implantation and could cause detrimental effects (Malizia et al., 1984).

PLGA is a very well-established synthetic biodegradable copolymer of poly lactic acid (PLA) and poly glycolic acid (PGA). PLGA has been widely used in a variety of medical devices, such as sutures and meshes, and is also used as a component of other device/pharmaceutical applications for sustained delivery of cells, proteins, peptides, and nucleic acids. It is widely as an inactive component of implanted depots; including, for example, Nutropin Depot^®^, Zoladex^®^ implant, and Lupron Depot^®^. Microparticles composed of PLGA and loaded with active agents have an excellent track record for safety and biocompatibility when implanted into soft tissues (Keshaw et al., 2009; Swider et al., 2018). However, as the performance of TIPS microparticles implanted into the tissue cavity left by the resected prostate gland has not been characterized previously, the effect of implantation will require further evaluation. This will include demonstrating it does not lead to increased fibrosis or less healing and potentially poorer recovery of epithelization of the anastomosis and recovery of nerves/muscle/vasculature that normally occurs over the first weeks and months post-surgery.

A facile and robust method was used to load the prepared TIPS microparticles with DTX. This approach offers advantages over other microsphere drug-device fabrication techniques, such as the solvent-emulsion evaporation process used to produce PACLIMER^®^ microspheres, including avoiding exposure of the chemotherapeutic agent to solvents during the fabrication process, and was capable of achieving high drug loading efficiencies. Moreover, preparation of the drug-device combination immediately prior to use allows the PLGA TIPS microparticles to be stored in a dry format, thus increasing their shelf-life by avoiding degradation.

Drug-device combination products present complex regulatory and scientific considerations. According to Article 1 (4) Medical Device Directive (MDD, 93/42/EEC) and Article 1 (4) Active Implantable Medical Device Directive (AIMDD, 90/385/EEC), incorporation of the chemotherapeutic agent with the TIPS microparticles, via physically combining them at the time of administration to the patient so that it becomes an integral part of the combination product, would classify the drug delivery system as a medical device incorporating, as an integral part, a medicinal substance with ancillary action.

The presence of DTX crystals on the surface of the TIPS microparticles indicates that DTX was loaded on the surface instead of by entrapment in the particles. DTX crystals observed on the TIPS particles were likely formed by anti-solvent precipitation and grew during the drying process as a result of solvent evaporation. In anti-solvent precipitation, super-saturation is achieved by the addition of the organic solution of drug to an anti-solvent, lowering the solubility of drug and leading to nucleation and crystallization of the drug (Park & Yeo, 2012). Therefore, DTX was solubilized in ethanol and then mixed with an anti-solvent (water) containing the TIPS microparticles. The hydrophobic and porous nature of the PLGA TIPS microparticles provided a suitable surface for DTX precipitation as it facilitated nucleation of drug and thus promoted crystallization on the surface, accounting for the high drug loading efficiency of ∼80%. The maximum loading onto the TIPS particles occurred within 60 minutes without the utilization of heating and cooling steps, making it a feasible time-frame for preparing the drug-device combination at the point of care. As DTX was loaded on the surface of the microparticles, the release of DTX is predominately controlled by DTX crystal dissolution instead of via degradation of PLGA polymer. The crystals of DTX slowly dissolved with time, maintaining the concentration gradient for diffusion and resulting in a slow release over five days. This is beneficial since the drug-device combination will be implanted into the tumor resection site where its prolonged localized release will help eradicate residual or shed tumor cells released during prostatectomy, minimizing the chance of recurrence. The amount of DTX released into perfusate during the first five days of the DTX release assay, simulating release and local activity *in vivo*, was sufficient to achieve 100% toxicity against the PC3 cells in the colony forming assay and corresponded with approximately 95% of the DTX being released from the microparticles. DTX release from the microparticles continued beyond five days with the quantity of DTX present in the perfusates collected at daily intervals up to day 10 continuing to show mitotic arrest despite being below detection threshold of UV spec. PC3 prostate cancer cells incubated in the perfusates collected at later time points were reduced in number and showed phenotypic features typical of apoptosis. Further studies are now warranted to demonstrate the efficacy of the DTX-TIPS microparticle combination as anti-cancer strategy using suitable *in vivo* models.

In conclusion, we have devised a novel drug-device combination that could be used in a new approach for adjuvant chemotherapy following radical prostatectomy. Future studies investigating the efficacy of the chemotherapy agents sustained release profile *in vivo* will be required to determine whether further optimization of the drug loading and release is required before progressing to clinical investigations.
